# Progressive Plantar Foot Pain as a Presentation of Arteriovenous Malformation: A Case Report of Diagnostic Delay

**DOI:** 10.7759/cureus.117941

**Published:** 2026-10-14

**Authors:** Han Wei Zheng

**Affiliations:** 1 Emergency Medicine, Thomas Jefferson University Hospital, Philadelphia, USA

**Keywords:** arterio-venous malformations, computed tomography angiography (cta), diagnostic delays, emergency medicine management, lateral plantar foot pain

## Abstract

Arteriovenous malformations (AVMs) of the foot are rare vascular anomalies that may present with nonspecific symptoms and mimic common emergency department conditions. We report a 26-year-old man with progressive plantar foot pain and swelling who had multiple healthcare encounters before an AVM was diagnosed. Initial evaluation included unremarkable radiographs, treatment for presumed cellulitis, and negative venous duplex ultrasonography for deep venous thrombosis. Persistent and worsening symptoms ultimately prompted targeted ultrasonography, which identified a plantar AVM. On subsequent emergency department presentation for severe pain and inability to bear weight, examination demonstrated a warm, exquisitely tender plantar lesion with pulsatile Doppler flow, and computed tomography angiography confirmed the diagnosis. The patient underwent transarterial embolization with subsequent improvement in pain, swelling, and ambulation. This case highlights the importance of reconsidering the differential diagnosis in patients with persistent or recurrent symptoms, particularly when clinical features are atypical for common conditions such as cellulitis or when abnormal vascular findings are present.

## Introduction

Arteriovenous malformations (AVMs) are high-flow vascular anomalies characterized by multiple direct communications between arteries and veins without an intervening capillary bed. They are vascular malformations that may be present at birth but can remain clinically silent and present at any age. AVMs may progressively enlarge over time, although the rate of progression is variable. Clinical manifestations vary according to lesion size, location, and hemodynamic characteristics and may include pain, swelling, warmth, skin discoloration, pulsatility, ulceration, bleeding, and functional impairment [1-3]. Symptoms of a plantar AVM may overlap with more frequently encountered emergency department conditions, including cellulitis, abscess, plantar fasciitis, stress injury, soft-tissue injury, and deep venous thrombosis [4]. Symptom onset may be due to progressive lesion enlargement and hemodynamic changes [5]. Accurate classification of vascular malformations is important because AVMs are distinct from low-flow venous malformations and have different clinical and therapeutic considerations [6].

Peripheral AVMs are uncommon and can be particularly challenging to recognize when they occur in anatomically unusual locations such as the foot [7]. This overlap may lead to an initial evaluation focused on more common diagnoses, particularly when the presenting symptoms are limited to pain, swelling, erythema, or localized tenderness. Plain radiographs may be unrevealing because they do not directly characterize vascular abnormalities, while targeted Doppler ultrasonography and cross-sectional vascular imaging can provide additional information regarding vascularity, flow characteristics, lesion extent, and vascular anatomy. Persistent or progressive symptoms despite appropriate treatment should therefore prompt reconsideration of the initial diagnosis and consideration of less common vascular etiologies. This case highlights the diagnostic challenges associated with plantar AVMs and emphasizes the importance of maintaining a broad differential diagnosis in patients with persistent or progressive foot pain and swelling.

## Case presentation

A 26-year-old man with no significant past medical history presented to the emergency department with worsening pain and swelling involving the plantar aspect of his left foot. Symptoms began approximately two months earlier while running. He denied a history of trauma, penetrating injury, fever, chills, or other systemic symptoms. Following symptom onset, the patient was evaluated by his primary care physician. Radiographs of the left foot demonstrated no acute fracture or other osseous abnormality. Conservative management was recommended. Over the subsequent month, pain and swelling progressed, prompting emergency department evaluation. Physical examination at that time was notable for plantar foot tenderness and erythema. Given concern for cellulitis, the patient was prescribed a five-day course of cephalexin and discharged.

Despite completing antibiotic therapy, symptoms continued to worsen. He subsequently returned to the emergency department because of persistent pain and swelling. Venous duplex ultrasonography demonstrated no evidence of deep venous thrombosis. He was later evaluated by orthopedic surgery in the outpatient setting, where conservative management was recommended. As symptoms continued to progress, the patient sought podiatric evaluation. Targeted ultrasonography of the symptomatic area demonstrated findings concerning for an AVM involving the plantar aspect of the left foot. The ultrasound was performed at an outside facility; therefore, the images were not available for review. The patient was referred for outpatient interventional radiology evaluation.

Before specialist consultation could occur, he returned to the emergency department because of severe pain that had progressed to the point that he was unable to bear weight on the affected extremity. Vital signs demonstrated a temperature of 98.6°F, heart rate of 108 beats per minute, respiratory rate of 20 breaths per minute, blood pressure of 142/79 mmHg, and oxygen saturation of 99% on room air. Physical examination revealed palpable dorsalis pedis and posterior tibial pulses. An ovoid region of pallor was present along the lateral plantar aspect of the left foot (Figure 1).

**Figure 1:**
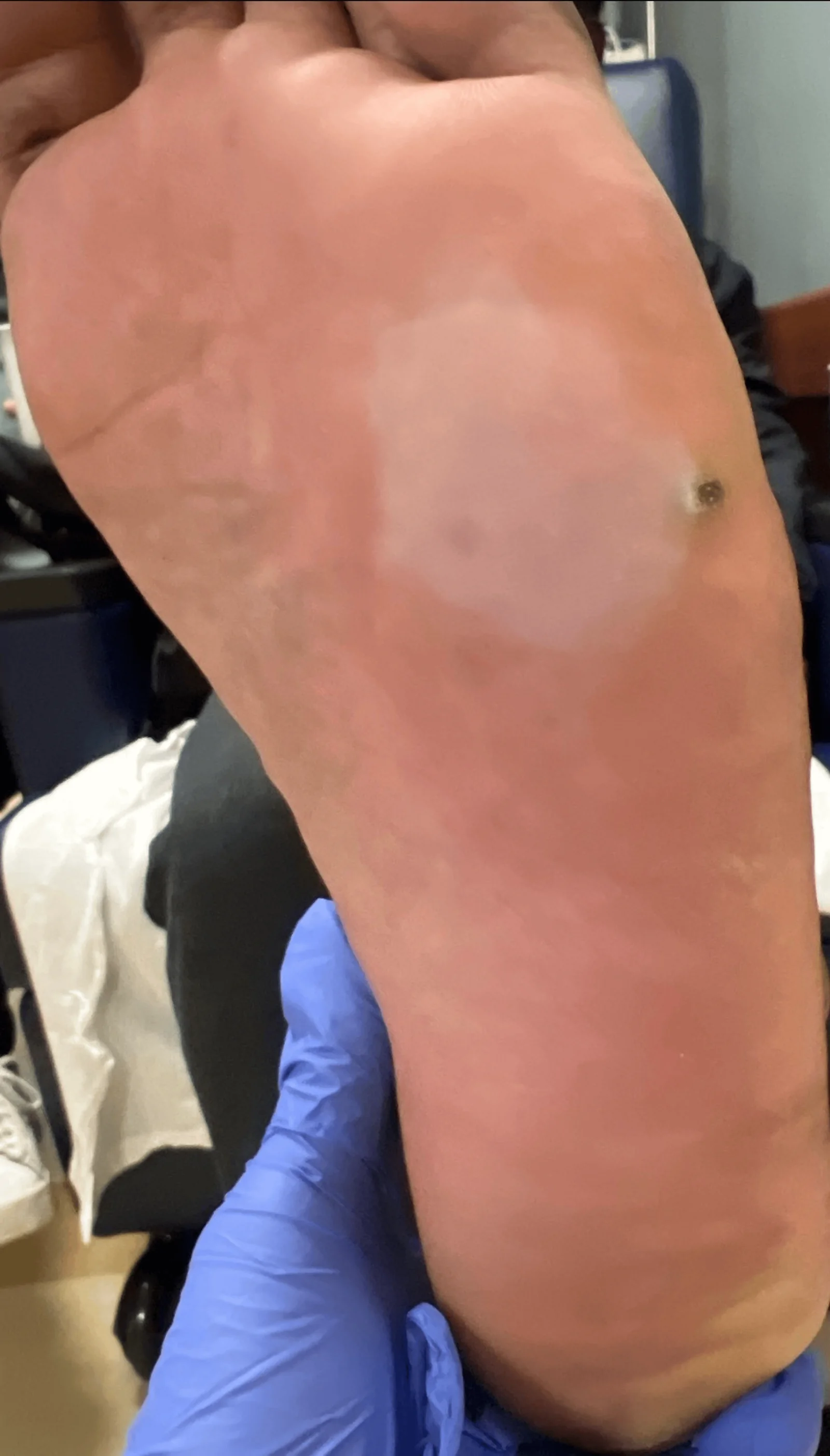
Clinical photograph of the plantar aspect of the left foot.

The area was warm and exquisitely tender to palpation. The dark lesion adjacent to it is an old callus. Bedside Doppler examination demonstrated pulsatile flow within the affected area. Interventional radiology was consulted from the emergency department and recommended computed tomography (CT) angiography. Imaging confirmed an AVM at the location of the pain (Figure 2). The CT angiography shows the posterior tibial artery and plantar arteries leading up to a 24.8 x 16.0 mm nidus of blood vessels along the plantar aspect of the foot with an extensive amount of early draining veins in the foot.

**Figure 2:**
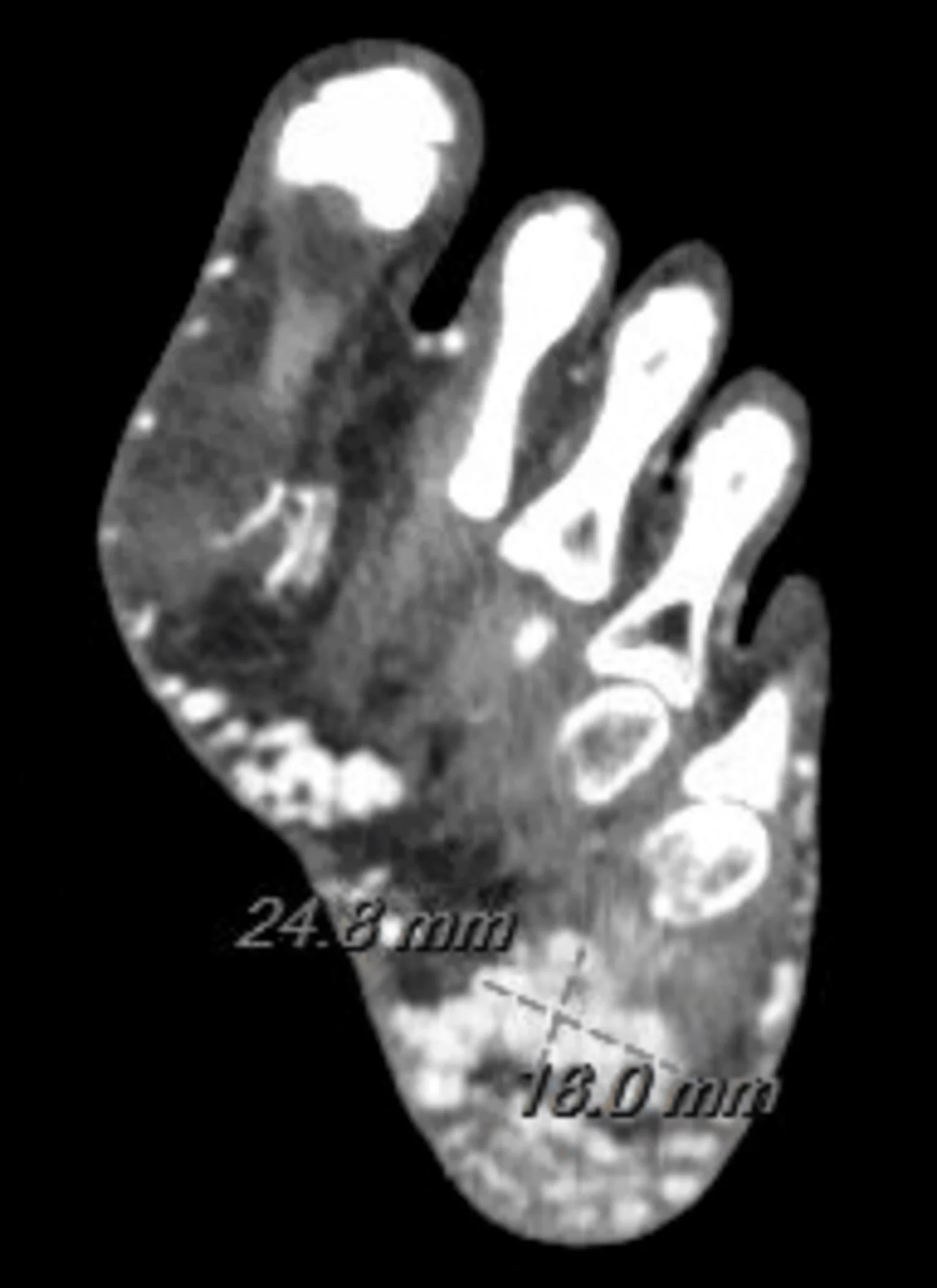
Computed tomography (CT) angiography demonstrates an abnormal cluster of enhancing vascular structures within the plantar foot, measuring approximately 24.8 × 16.0 mm, consistent with a plantar arteriovenous malformation.

The patient was admitted to the hospital for definitive management and subsequently underwent transarterial embolization. A wire and catheter were advanced into the distal left posterior tibial artery, where arteriography demonstrated the AVM nidus. The lesion was subsequently embolized using N-butyl-2-cyanoacrylate. The patient was instructed to follow up in one month. At outpatient follow-up, he reported significant improvement in pain and swelling with restoration of ambulation.

## Discussion

The diagnostic course in this case is consistent with previously described challenges in recognizing peripheral AVMs. The patient's initial plantar pain, swelling, and erythema led to treatment for presumed cellulitis. Despite these interventions, his symptoms continued to progress. Plantar foot AVM may be initially incorrectly diagnosed, highlighting the potential for delayed recognition of these lesions when they present with nonspecific symptoms [8]. High-flow vascular malformations may demonstrate increased local temperature, pulsatility, bruit, thrill, or arterialized venous flow because of abnormal arteriovenous shunting [9]. Similarly, Tanaka et al. reported two cases of peripheral micro-arteriovenous fistulas that were initially diagnosed as refractory cellulitis because of overlapping findings including redness, warmth, tenderness, and edema [10]. Although micro-arteriovenous fistulas are distinct from AVMs, these cases demonstrate how peripheral high-flow vascular abnormalities may mimic common inflammatory or infectious conditions. This case further emphasizes the importance of reconsidering the differential diagnosis when symptoms fail to improve with appropriate treatment.

Physical examination may provide important clues to a vascular etiology. In the present case, the lesion was warm and exquisitely tender, and bedside Doppler examination demonstrated pulsatile flow. The presence of pulsatile flow within a painful plantar lesion should therefore increase suspicion for a vascular abnormality, particularly when infectious or musculoskeletal diagnoses do not adequately explain the clinical course. Careful examination of the symptomatic area, including assessment for abnormal vascular flow, may provide an important diagnostic clue before advanced imaging is obtained.

Imaging plays a central role in the diagnosis and characterization of peripheral vascular malformations. Doppler ultrasonography is an important noninvasive modality for superficial lesions and can characterize vascular channels and flow dynamics [11]. Madani et al. noted that ultrasound is frequently the first imaging study used to evaluate peripheral vascular malformations and is particularly useful for small or superficial lesions involving the feet and hands [7]. In the present case, targeted ultrasonography ultimately identified the plantar AVM after initial radiographs were unrevealing and venous duplex ultrasonography had excluded deep venous thrombosis. This distinction is clinically relevant because a venous duplex examination performed primarily to evaluate for deep venous thrombosis may not fully characterize a focal vascular malformation unless the symptomatic area is specifically interrogated.

Cross-sectional imaging can further define lesion extent and vascular anatomy. MRI is particularly useful for characterizing the extent of vascular malformations and their relationship to surrounding soft tissues, while CT angiography can rapidly demonstrate feeding arteries, the nidus, and early venous drainage when a high-flow lesion is suspected. The American College of Radiology considers duplex Doppler ultrasonography, MRI/MR angiography, and CT angiography appropriate imaging options for suspected extremity vascular malformations, with the choice dependent on the clinical scenario and purpose of imaging [11]. In the present case, CT angiography was obtained after abnormal vascular flow and persistent severe symptoms raised concern for an AVM and confirmed the diagnosis. Thus, the progression from targeted ultrasonography to CT angiography illustrates the complementary role of noninvasive and cross-sectional vascular imaging in patients with suspected peripheral AVM.

The plantar location of the lesion is particularly relevant because AVMs of the foot are uncommon and may produce substantial pain and functional impairment. Yakes et al. reported three patients with AVMs involving the plantar aspect of the foot who were successfully treated with minimally invasive embolization. Their report emphasized the importance of defining AVM angioarchitecture to guide treatment selection and described complex plantar lesions involving different arterial and venous configurations [12]. Yuzaki et al. subsequently reported a 24-year-old man with a plantar AVM who presented with pain and difficulty walking. Because surgical resection was considered likely to result in functional impairment, the patient underwent multiple embolization procedures with subsequent resolution of symptoms [8]. These findings are consistent with the present case, in which progressive plantar pain ultimately resulted in inability to bear weight and definitive treatment with transarterial embolization was followed by substantial improvement in pain, swelling, and ambulation.

Treatment of peripheral AVMs is individualized according to lesion architecture, flow characteristics, location, symptoms, and the feasibility of achieving durable eradication of the nidus. Imaging is important not only for diagnosis but also for treatment planning. Hyodoh et al. emphasized that Doppler ultrasonography, CT, and MRI can help establish the diagnosis, determine lesion extent, and guide treatment, while management of high-flow lesions often requires a multidisciplinary approach [9]. Madani et al. similarly emphasized detailed imaging and multidisciplinary management for complex peripheral vascular malformations [7]. Contemporary imaging literature continues to support a multimodality approach incorporating Doppler ultrasonography, MRI/MR angiography, CT angiography, and catheter angiography when appropriate for treatment planning [13].

Endovascular embolization is an established treatment option for symptomatic peripheral AVMs and may be particularly valuable for lesions in anatomically challenging locations. Yakes et al. demonstrated successful minimally invasive embolization of plantar AVMs, while Yuzaki et al. reported successful eradication of a complex plantar AVM using embolization and sclerotherapy [8,12]. The present patient's improvement following transarterial embolization is consistent with these reports. However, the literature also demonstrates that treatment strategies must be individualized, as lesion architecture and involvement of surrounding structures can substantially influence the choice between embolization, sclerotherapy, surgery, or combined approaches [13].

## Conclusions

This case reinforces the importance of diagnostic reconsideration in patients with persistent or recurrent foot pain and swelling who do not respond as expected to treatment for common conditions. AVMs of the foot are uncommon but important causes of persistent pain and swelling. Because their clinical manifestations may mimic cellulitis, abscess, and other common conditions, they can present a diagnostic challenge and lead to delayed recognition. Emergency physicians should consider AVMs in patients with refractory symptoms, recurrent presentations, atypical features inconsistent with cellulitis or abscess, or abnormal vascular findings on examination. Early vascular imaging and specialty consultation can facilitate timely diagnosis and definitive treatment, potentially reducing the risk of persistent pain and functional disability.

## References

[1] Mulliken JB, Glowacki J (1982). Hemangiomas and vascular malformations in infants and children: a classification based on endothelial characteristics. Plast Reconstr Surg.

[2] (2026). International Society for the Study of Vascular Anomalies. ISSVA classification for vascular anomalies. https://www.issva.org/classification.

[3] Vaidya S, Cooke D, Kogut M, Stratil PG, Bittles MA, Sidhu M (2008). Imaging and percutaneous treatment of vascular anomalies. Semin Intervent Radiol.

[4] Flors L, Leiva-Salinas C, Maged IM (2011). MR imaging of soft-tissue vascular malformations: diagnosis, classification, and therapy follow-up. Radiographics.

[5] Kohout MP, Hansen M, Pribaz JJ, Mulliken JB (1998). Arteriovenous malformations of the head and neck: natural history and management. Plast Reconstr Surg.

[6] Lee BB, Baumgartner I, Berlien P (2015). Diagnosis and treatment of venous malformations. Consensus document of the International Union of Phlebology (IUP): updated 2013. Int Angiol.

[7] Madani H, Farrant J, Chhaya N, Anwar I, Marmery H, Platts A, Holloway B (2015). Peripheral limb vascular malformations: an update of appropriate imaging and treatment options of a challenging condition. Br J Radiol.

[8] Yuzaki I, Aramaki-Hattori N, Tamura M (2020). Arteriovenous malformation on the sole of the foot treated successfully by embolization. Radiol Case Rep.

[9] Hyodoh H, Hori M, Akiba H, Tamakawa M, Hyodoh K, Hareyama M (2005). Peripheral vascular malformations: imaging, treatment approaches, and therapeutic issues. Radiographics.

[10] Tanaka M, Serizawa F, Nagaoka Y (2018). Two cases of micro-arteriovenous fistula in the lower extremity with misdiagnosis of refractory cellulitis. Ann Vasc Dis.

[11] Obara P, McCool J, Kalva SP (2019). ACR Appropriateness Criteria® clinically suspected vascular malformation of the extremities. J Am Coll Radiol.

[12] Yakes W, Huguenot M, Yakes A, Continenza A, Kammer R, Baumgartner I (2016). Percutaneous embolization of arteriovenous malformations at the plantar aspect of the foot. J Vasc Surg.

[13] Schmidt VF, Masthoff M, Czihal M (2021). Imaging of peripheral vascular malformations-current concepts and future perspectives. Mol Cell Pediatr.

